# Predicting Hunting Trends: The Effect of Pelt Prices, Urbanization and Weather on the Hunting/Trapping of Twelve Small Fur-Bearing Animals in Wisconsin, 1930–2018

**DOI:** 10.3390/ani16091392

**Published:** 2026-05-02

**Authors:** Michael J. Lynch, Leo J. Genco

**Affiliations:** 1 Department of Criminology, University of South Florida, Tampa, FL 33620-8100, USA; 2 Department of Criminal Justice, Tarleton State University, Stephenson, TX 76402, USA; lgenco@tarleton.edu

**Keywords:** hunting trends, animal wellness, human wellness, human dimension of wildlife, macro-correlates of hunting, pelt prices and hunting, urbanization and hunting

## Abstract

This study examines structural features that may impact the hunting/trapping of small, fur-bearing animals over time. The prior literature suggests a relationship between species, ecological and human well-being that renders hunting trends of social interest. In that view, factors affecting hunting trends have policy relevance related to how species maintenance/health affects ecosystems and human well-being. This study employs data on twelve fur-bearing species taken by hunting/trapping in Wisconsin from 1930 to 2018. Prior research has identified several predictors of hunting trends (e.g., pelt prices, snow and rainfall amounts, temperature, gasoline prices and urbanization). Controlling for the effect of the prior level of hunting, few exogenous factors predicted species-specific hunting trends in Wisconsin. Macro-factors predicted that hunting trends are outside the control of wildlife regulators and may require innovative strategies to address. The results suggest that some omitted processes may be better predictors of hunting trends.

## Simple Summary

Extant research suggests that wildlife health and density have links to ecosystem health and also to human health. Those studies suggest that maintaining healthy wildlife populations has benefits for humans. In various wildlife and ecological studies, wildlife health has also been linked to hunting, which can have both positive and negative effects on the preservation of wildlife populations. Understanding factors that affect the health of wildlife populations is useful to wildlife managers and for constructing wildlife regulations and preservation policies. This article examined whether the hunting/trapping trends for 1930–2018 for twelve small fur-bearing species in the US state of Wisconsin could be predicted using previously identified and newly proposed large scale (i.e., structural) measures of land use (e.g., federal and state forest acres, urbanization), weather (temperature, snowfall and rainfall), and economic indicators (e.g., gasoline prices and species-specific pelt prices). For most species, the best predictor of current hunting/trapping was prior hunting and trapping. Other predictors that had statistical effects included urbanization and pelt price, but these effects were only seen for five of the twelve species.

## 1. Introduction

The current study examines macro-level factors that may influence hunting/trapping trends over time (1930–2018) using data from the state of Wisconsin. Understanding hunting trends is important for crafting policies affecting nonhuman species concentrations and wellness, and ecological wellness [1]. The related literature indicates that nonhuman species' well-being also impacts human health and wellness as well (see discussion below). Consequently, factors affecting nonhuman species’ health and wellness can have feedback effects that impact ecosystem, ecological and human health and wellness. One way that species’ wellness—an idea that includes broader measures of species’ health such as dispersion, concentration, prevalence, and biodiversity indicators—may be affected is through hunting. Though we discuss the link between species, ecosystem and human wellness below as a rationale for this study, our work does not measure the effect of hunting on species’ wellness indicators since we do not have species population estimates for the 89 years of data included in our analysis, and we only have data on the number of species taken by hunting and trapping. Rather, our research attempts to determine if there are larger, macro-level forces that affect hunting trends, and whether knowing about those forces can assist in promoting species’ wellness.

Hunting/trapping has been examined in relation to several concerns in the relevant literature, such as: (1) the rights of wildlife to be protected from harm [2]; (2) the use of hunting to maintain “healthy” wildlife population parameters [3]; (3) the intersection of hunting with economic livelihoods and maintenance of sufficient wildlife populations to support recreational hunting [4]; (4) the provision of protein in the diets of the rural poor [5]; and (5) the rights and needs of indigenous peoples related to hunting [6]. These are all important concerns, and regardless of how one views hunting philosophically, it has importance from several different perspectives. Our work does not address specific hunting-related philosophical approaches, but rather addresses the question of whether or not the patterns in hunting/taking of animals can be predicted using macro-level variables and trends. If such relationships exist, they may be important in wildlife conservation management, research and policy. Researchers, policymakers, animal rights activists, hunters and the public may all have an interest in the effect of hunting on wildlife wellness, and some of these groups may also be interested in the intersection between wildlife, ecosystem and human health.

Hunting/trapping participation and wildlife taking data are typically collected using post-hunting phone or mail surveys, or through the use of field counts of animals taken. Few of those studies, however, examined factors that may affect trends in the taking of animals via hunting/trapping over time [7]. As prior studies suggest, these factors can include weather, climate change, trends in deforestation, land use patterns, urbanization, or economic conditions. The effects of these broader macro-level forces on hunting trends have not been widely examined. As noted, identifying macro-level factors affecting hunting/trapping trends may be useful to policy makers considering wildlife protection and management plans related to, for example, forest protection or other forms of development planning and hunting regulations, including species-specific bag limits.

It should be noted that in undertaking this study, it is not our intention to make value judgments about hunting/trapping, but more simply to explore various factors that may affect the level/trend in hunting over time, and to explore how animal taking via hunting/trapping may be impacted by social, economic and ecological factors. Obtaining a broader understanding of factors affecting hunting/trapping trends and levels over time potentially has relevant policy implications related not only to species’ wellness and health, but also to ecological and human health and wellness as well.

## 2. Background

### 2.1. The Intersection of Species and Human Wellness

Wildlife and environmental conservation have become increasingly important concerns in the contemporary world, especially as human development expands and encroaches further on ecosystems, and as the adverse effects of climate change on ecosystems accumulate. The health of wildlife and natural environments is important in itself, but also has a relationship to human health and well-being [8]. Healthy and sustainable wildlife populations and ecosystems promote biodiversity richness, and successful management of these systems can promote human health [9]. In 2015, this intersection was recognized in a study published by researchers from the Rockefeller Foundation that drew attention to the concept of “planetary health” [10]. The researchers defined planetary health as including “the health of human civilization and the state of the natural systems on which it depends” (p. 1978). In discussing this intersection, Krisanti et al. [11] noted that biodiversity wellness is affected by how humans use and protect ecosystems, meaning that there is a nonrecursive relationship between wildlife and human health.

Studies suggest that one challenge to the maintenance of ecosystems and wildlife biodiversity wellness may be hunting [12]. Some studies suggest that “appropriate levels” of species harvests can have beneficial impacts on species wellness and biodiversity [13,14]. But, while some studies favor appropriate animal harvesting [15], others suggest that hunting/harvesting wildlife may adversely affect wildlife populations [16], limit the genetic diversity of a species’ population [13], and also negatively impact other wildlife species in ‘hunting-improved’ ecosystem habitats, or ecosystem locations where extensive hunting of certain species occurs [17]. These observations about the hunting–biodiversity relationship may be a product of studies that have examined a limited array of species (e.g., keystone species and trophy species impacts versus hunting in a given location of an overpopulated herbivore species, such as deer, see endnote 1), and further research is needed to adequately address this hunting-effect debate.

### 2.2. Hunting, Biodiversity, and Hunting Rights and Animal Rights

Conclusions concerning the impact of hunting on species biodiversity have been potentially limited by the scope of that research—that is, by the number of different species that have been examined to assess the biodiversity impacts of hunting. Conclusions related to the impact of hunting on species biodiversity may also be constrained by the absence of research employing longer-term species population data. Longer-term species population data, however, which became more widespread in the late 20th century, are biased toward certain species and have been described as haphazard and containing numerous methodological deficiencies [18].

Consistent with the above observations, few studies have examined long-term trends or a broad cross-section of empirical data on hunted species and biodiversity impacts [19,20]. Thus, relatively little empirical research exists upon which to reach informed decisions about the effect of hunting on wildlife populations, particularly in the US, and especially outside of species of concern such as keystone species (e.g., cougars, bears, deer, wolves [21]). Although the current study does not measure the effect of hunting on species wellness, biodiversity or population size, the literature is relevant because it draws attention to the association between hunting and species wellness.

Many studies address the juxtaposition of hunting rights and animal rights. The literature illustrates concern related to the impact of hunting on the size and well-being of wildlife populations for different reasons. Often, those arguments are drawn from philosophical rationale. When data has been employed to address the intersection of species wellness, hunting and animal rights, those discussions tend to rely upon limited data and analysis. Consequently, some suggest that these limitations have promoted conjecture and hypothetical assumptions to be made about the intersection of animal rights, hunter rights, and species wellness [22].

Beneath questions concerning the relationship between species, ecosystem and human wellness are questions about factors that may influence trends in hunting and the taking of animals. That issue is important for the following reason—if species wellness and human health are related, and hunting damages species wellness, it could also have adverse human wellness consequences (and one could argue the reverse could also be true if hunting promotes species wellness). At the same time, the consequences of hunting for wildlife species that exist outside the wildlife–human intersection should not be ignored (e.g., the ethics of hunting; ways in which hunting harms wildlife, or produces pain; species rights, etc.). Nevertheless, if wildlife species’ and human species’ wellness are interconnected, this would imply a need to potentially control hunting if or when hunting produces adverse wildlife species impacts. To our knowledge, however, there is no current research that identifies an objective empirical standard indicating when a species should be protected to promote ecosystem or human health and wellness—and it is not our intention to propose that such a measure can be easily created, nor will one be proposed here. All that can be said in this regard, consequently, is that some level of hunting may potentially damage species, ecosystem and human health and wellness. Tracking hunting outcomes, such as the number of animals harvested within a species, as many states do with particular species (i.e., on deer [7]), may help provide data that can be employed to initiate further examinations of this kind of question.

### 2.3. Factors Influencing Hunting Trends

Hunting trends have sometimes been employed to predict wildlife species population size and growth trends [23,24], and to assist in assessing species wellness, or the need for hunting control policies. The simplest explanation for the relationship between hunting and the number of species taken is that it is primarily determined by the number of hunting licenses sold, though other factors may also contribute to this relationship, such as natural declines in wildlife species reproductive cycles [25], which in turn is affected by other macro-level trends such as climate change [26]. It is therefore useful to examine how other factors outside of licenses sold predict hunting trends over time, since these factors may have policy relevance that promotes ecosystem, animal, and human wellness.

Exploration of attitudes towards hunting and animals is one way to explore this issue [27,28,29]. Surveys related to hunting and animal welfare and animal rights provide information that informs conservation-based research, such as conservation research in criminology used to devise wildlife policy [30]. The potential relationship between economic, social and ecological contexts associated with hunting trends, however, has not been widely examined in the related literature. This observation raises the question of whether certain social, economic, or environmental changes outside the control of wildlife managers might impact hunting trends and tendencies. For example, it is possible that weather/climate, such as warmer or wetter seasons, may affect hunting volume by creating conditions unfavorable to hunting; or that climate/weather may produce adverse conditions that limit animal mating and offspring rates and rearing effectiveness, limiting hunting opportunities through restricted animal availability [31]. In short, there are various pathways through which climate/weather may affect the hunting of species generally, or the hunting of specific species. And although climate/weather is not something policy makers can control, they still need to understand how it affects animal reproduction, hunting and the taking of species, since this could impact other dimensions of hunting control policies they can affect.

## 3. The Current Study

Building upon the diverse array of observations summarized above, this study employs 89 years (1930–2018) of hunting/trapping data from the state of Wisconsin to examine whether structural features of the economic, social and ecological systems in Wisconsin are empirically related to hunting/trapping trends. We examined hunting trends for twelve species defined as “fur-bearing game” in that data. We used fur-bearing game data since fur-bearing species hunting/trapping, and the effects of climate and weather have been examined in several previous studies [32,33]. Prior research indicates that pelt prices may be related to hunting/trapping levels for fur-bearing species, and pelt price is an economic market variable that can be measured to assess its effect on hunting trends [34,35,36,37,38,39]. In addition to pelt price, we included the following additional variables that might affect the number of animals taken by hunting/trapping: (1) climate/weather, which included measures of (A) mean temperature, (B) rainfall and (C) snowfall; (2) percent of the population living in urbanized areas; (3) gasoline prices, which can impact the financial ability of people to hunt frequently; and (4) broader measures of economic health that address the effect of recession/depression on hunting (see discussion below). In preliminary estimates not included here (see discussion below), we also assessed the impacts of fourteen additional macro-level variables (e.g., acres of land in, and number of state, federal and local parks measured as separate variables and in additive forms) on hunting and trapping trends. None of these latter variables were significantly related to the outcomes, and in the models presented below, we excluded those variables after initial analysis to simplify the discussion of relevant results.

## 4. Materials and Methods

The dependent variable is a rate variable. We transformed counts of twelve different fur-bearing species trapped or taken by hunting, 1930–2018, each of which is assessed independently, into rates measured as species taken relative to the human population, which also controlled for the influence of population size on hunting/trapping. These data were extracted from the Wisconsin Wildlife Harvest Summary, 1930–2018 [40]. These data represent a combination of data from several sources, and include data from hunter surveys and other procedures employed by each of the following groups from which data were collected and synthesized by state of Wisconsin officials: Wisconsin Department of Natural Resources; U.S. Fish & Wildlife Service; Great Lakes Indian Fish & Wildlife Commission; U.S. Forest Service; U.S. Department of Agriculture Animal, Plant and Health Inspection Service; Wisconsin County Foresters; and university studies. As noted in the literature, estimates of species taken by hunting can be made using a variety of techniques, but often rely on self-reporting, which can be biased, but is often employed due to cost-effectiveness [41]. No data collection method for animals taken is immune from bias and errors in the estimate of animals taken, and estimates of the number of species taken can be influenced by several factors, some of which can be corrected [42]. We cannot, however, attest to the limitations or adequacy of the methods used by the state of Wisconsin that were employed to create these data.

*Dependent variables.* The dependent variables in this study were species-specific rates of animals reported taken by hunting or trapping, standardized by human population. Although the official data included information on fourteen fur-bearing species, we eliminated two species—the badger (*Taxidea taxus*) and fisher (*Pekania pennanti*)—due to shorter-term trend data availability for these species. Badger hunting was discontinued in Wisconsin from 1954 onward, and the badger was later (1955) placed on the Wisconsin protected species list. Consequently, predicting the trend in badger hunting/trapping has little current policy relevance. Fisher counts were also truncated relative to the entire series and were not recorded until the late 1980s. The twelve included species and years were as follows: (1) muskrat (*Ondatra zibethicus*, 1930–1987, 1990–2018); (2) raccoon (*Procyon lotor*, 1930–1988, 1990–2018); (3) mink (*Neogale vision*, 1930–1988, 1991–2018); (4) red fox (*Vulpes vulpes*, 1932–1956, 1959–1988, 1991–2018); (5) gray fox (*Urocyan cinereoargenteus*, 1932–1956, 1959–1988, 1991–2018); (6) beaver (*Castor canadensis*, 1933–1938, 1940–1943, 1945–1988, 1990–2018); (7) river otter (*Lontra canadensis*, 1930–1946, 1948–1953, 1955–2018); (8) bobcat/Lynx (*Lynx canadensis*, 1930–1956, 1959–1963, 1970–2018); (9) coyote (*Canis latrans)* and gray wolf (*Canis lupus*, 1930–1956, 1959–1963, 1965–1988, 1991–2018); (10) the short-tailed weasel (*Mustela richardsonii*), the long-tailed weasel (*Neogale frenata)*, and the least weasel (*Mustela nivalis*, 1917–1920, 1927–1959, 1970–1988, 1991–2018); (11) opossum (*Didelphis virginiana*, 1927–1959, 1961–1988, 1991–2018); and (12) skunk (*Mephitis mephitis*, 1927–1959, 1961–1988, 1991–2018). As noted, the number of a species trapped/hunted was standardized against the human population, and measured as the number of animals killed/trapped per 100,000 human population. This standardization technique is a useful means to control for the potential effect of human populations on the number of animals taken by hunting and trapping.

*Independent variables.* A number of independent variables measuring environmental, economic and ecological factors were examined to ascertain their potential impact on wildlife taking. Several variables examined in initial models (analysis not shown here; specific variables shown below) proved inefficient (i.e., they had very low r values, and/or were insignificant in initial multivariate models) in predicting the outcomes and were excluded in the final models shown here (see Table 1). Those variables included various measures of land use and were chosen for initial analysis based on the hypothesis that changes in land use patterns over time could impact the availability of animal populations for hunting/trapping activities, and through this mechanism, would affect the trend in hunting. Specifically, land use variables included: (1) percent land in state parks; (2) percent land in federal parks; (3) percent land in forested areas; (4) percent land that was farmland; (5) percent land in urbanized areas; (6) a combined measure of 1–4; and (7) state population. Several factors representing combinations of these controls (e.g., 1 + 2; 1 + 2 + 4, etc.) were also assessed. None were efficient or significant in predicting species-specific hunting trends, and were dropped from further analyses.

Included independent predictors by year were as follows: (1) pelt prices for each species; (2) annual mean rainfall; (3) annual mean snowfall; (4) annual mean temperature; (5) gasoline price; (6) percent urban population; and (7) a variable coded no/yes for recession/depression (0 = no recession/depression). Pelt price data were extracted from the Wisconsin Harvest Summary report. Pelt price data was missing in some years, and was interpolated, and was also checked for reasonableness against other pelt price data sources, For example for gray fox pelt prices after 1965 for Arkansas see [43]. We applied an inflation adjustment to standardize pelt prices in 2018 dollars. Data on mean temperature, rainfall and snowfall were collected from the Wisconsin State Climatology Office [44]. Gasoline prices were collected from the US Energy Information Administration [45,46] and were then inflation-adjusted. The percentage of the population living in urban areas was extracted from the Iowa State University Community Indicators Program data [47], which has decennial data for each state.

Hypotheses related to the above predictors were as follows.

**H1:**  *Gasoline price (GAS$). Prior studies indicated that gasoline price has a negative impact on hunting* [34,35]*, so that as gasoline price increases, hunting declines.*

**H2:**  *Pelt Price (PELT$). Prior research indicates that pelt prices may affect hunting and trapping* [34,35,36,48]*, so that as pelt prices rise, hunting and trapping increase.*

**H3:**  *Weather conditions, Rainfall and Snowfall (Rain; Snow). Few prior studies have examined the effect of unfavorable weather conditions on hunting and trapping, with prior studies focused on nonfur-bearing animals* [49,50,51,52,53]*. Prior studies indicate that rainfall and snowfall may affect hunting outcomes as a result of decreased hunter participation during poor weather or increased hunter participation during favorable weather. Research also indicates that rainfall and snowfall may affect animal behavior. These results suggest that years with higher rainfall and snowfall amounts should have lower animal harvests.*

**H4:**  *Climate change, temperature (TEMP). Climate change is not only potentially associated with weather effects such as changes in rainfall and snowfall, but also with changes in temperature. To be sure, temperature is only one indicator of climate change, but here we include it as a measure of climate change to separate its effects from rainfall and snowfall effects. Changes in temperature may affect the behavior of animals, or affect species competition, availability and density* [33,54]*. In terms of a hypothesis, it is unclear whether temperature should increase or decrease hunting/trapping, though we hypothesize that an increase in temperature may have a positive effect on fur-bearing species breeding, and increase their prevalence, which could expand opportunities for successful species hunting and trapping.*

**H5:**  *Percent urban population (Urban%). Increased urbanization changes the volume of natural landscapes, fragments species' habitat, and affects the capability of landscapes to support wildlife population density and different wildlife species* [55]*. Landscape fragmentation associated with expanded urbanization provides major challenges to species biodiversity and population viability* [56]*, though these effects may be less harmful to more adaptive species* [57,58]*. We hypothesize that increased urbanization would have a negative effect on most fur-bearing animal harvests.*

Models were estimated using ordinary least squares regression. The data fit OLS assumptions: the independent variables were not multicollinear, and the error terms were normally distributed. The data represented the entire population of available measurements and were not based on a sample, so sampling issues were not present. Initial equation estimates, however, were found to be affected by autocorrelation, and initial estimates for most species had Durbin–Watson statistics that were not within acceptable limits. To address that problem, equations were corrected for autocorrelation using a lagged dependent variable as an independent predictor (i.e., the number of species Y taken in year X is related to the number of species Y taken in year X − 1). The lagged variable was specific to each species equation and was necessary for all fur-bearing animal models except for the gray fox model. The lagged variable procedure is appropriate when the autocorrelated error problem is related to first-order serial correlation [59]. In addition, the lagged dependent variable improves model fit and controls for omitted variable bias, and is based on an assumption that hunting observations in year X are related to the level in the preceding year [60].

## 5. Results

To begin, we note that all hunting trends were moderately to strongly predicted (R^2^ ranged from 0.482 to 0.856), with the exception of muskrat hunting (R^2^ = 0.08). In part, these well-fit models were affected by the inclusion of a lag of the dependent variable to correct for autocorrelation, except in the case of the gray fox model, which was not impacted by autocorrelation. Given the R^2^ results for these equations, we can have confidence that the significant variables are useful predictors of the outcomes. It should also be noted that the significance of the lagged variable in most models may also indicate that omitted variables might be useful for explaining the trend in hunting/trapping for the assessed species. Due to the large number of potential macro-level independent variables we assessed (i.e., including the variables omitted from the results provided here, the effects for twenty-one macro-level variables were assessed), it is difficult to propose other relevant macro-level variables that might be relevant to the trends examined here. One could, however, hypothesize that an important omitted variable is the number of hunting licenses issued in Wisconsin. Valid hunting license data counts, however, were only available for a very limited portion (from 2011 to 2018) of the time-series data we employed in this study. As one reviewer suggested, other relevant macro-level variables that might affect habitat size and quality and thus impact hunting/trapping include logging and mining. The reviewer also suggested that species-specific quotas and changing conservation status for some species could also potentially matter. We examined some of these additional variables to assess their availability. Logging and mining data for Wisconsin is limited, and is available sporadically, or for some counties but not all counties in Wisconsin, thus limiting our ability to create a state-wide measure of these variables. Using the “Appendix” from the Wisconsin Wildlife Harvest Summary, 1930–2018 [40], we concluded that there was no specific evidence of species-specific quotas for small fur-bearing game. The Appendix to that report did provide evidence of changing conservation status for some species in our data – but these changes were limited and affected only one year. Most changes in conservation status involved wolves, which were not included in our study. This change led to the suspension of hunting/trapping of wolves in some years. In these cases, the count for wolves would be “0” in certain years. Since our analysis treated “0” as missing, knowing the conservation status of wolves could help predict “0” count years, but not necessarily counts in other years, and would be expected to have little influence on predicting the long-term trend. Knowledge of wolf hunting could be important since restoring wolf populations could affect availability of some species in our study, and might be an additional control that could be introduced in future research.

Across the twelve species-specific models, few of the independent variables had consistent impacts (see results in Table 1). The two most important variables across species models were pet price (PELT$) and percent urban (URBAN%). PELT$ was significant and positive in five models (raccoon, skunk, weasel, gray fox and mink), indicating that for those species, an increase in PELT$ led to an increase in the number of species taken. This result indicates limited, species-specific support for hypothesis 1 (i.e., for five of 12 species examined). The effect of PELT$ is clearly a market effect that hunters might consider when planning to hunt or trap a specific species. It is difficult to suggest why PELT$ should affect the hunting of some but not other species. On the human side of the equation, drawing from economic arguments, one could argue that hunters of some species appear to act rationally by considering PELT$. It is also possible that for some species, the PELT$ effect is stronger because of greater fluctuations in PELT$ or higher PELT$ magnitudes, and hunters of those species pay closer attention to pelt prices because they are more likely to also sell pelts. Additional analysis of pelt price trends and comparisons across species would be needed to sort out this question, and future research is required to understand this effect more fully.

The second most common effect was for URBAN%, which was also significant in five models. In four models (otter, weasel, bobcat and gray fox), the relationship was negative, indicating that an increase in urbanization was related to a decline in hunting for those species. This result perhaps indicates that for these species, increased urbanization alters the spatial proximity of these species to potential hunters, perhaps affecting species availability for hunting, and the quality of ecosystems available to support those species. In addition, if urbanization is affecting the spatial dispersion of some species and driving them further from urban areas, this may affect hunters’ willingness to travel further to hunt those species. In one model (raccoon), the result was positive, indicating that as urbanization increased, raccoon-taking increased. This may be because raccoons, perhaps unlike other species, are less affected by and adapt more easily to urbanization [61].

The remaining variables had a small and varying impact on the prediction of hunting trends across the twelve species examined over time. For example, rainfall had only one significant impact, which was in the expected negative direction, for mink. In the case of mink, if rainfall were heavy, it could affect the behavior of both mink and hunters. As noted on one hunting enthusiast webpage (ontheoutside.co), it was stated that under conditions of heavy rainfall, near aquatic landscapes where mink live can become boggy and difficult for hunters to traverse, limiting hunters’ ability to successfully hunt mink. For mink, it was noted that heavy rainfall can decrease time spent outside dens, thus making them less available to hunters, producing a decline in hunting/trapping success.

Gas price (GAS$) had only one significant effect, and the effect was in the opposite direction from the hypothesized relationship in the gray fox model. A gas price effect has been noted in prior research for other species [34,35]. Why the gas price effect would only exist for gray foxes is difficult to explain. This effect could occur if gray foxes were located further from where fox hunters live, so that rising gasoline prices would, in this case, act to suppress gray fox hunting. This explanation, however, seems implausible. The habitats of gray fox and red fox overlap, so it is unclear why gasoline prices would affect gray fox but not red fox hunting/trapping patterns. It is possible that there is some form of complex interaction between gasoline prices and pelt prices that might also be driving these relationships that requires further investigation in future research.

Temperature also had a significant positive effect on the gray fox. It is unclear why temperature would have this limited species-specific effect. Temperature variations can certainly affect the behavior of any of the species examined here. Extant research indicates that rising temperatures associated with climate change, for example, affect the behavior of various species [26,33,54]. Those changing behavioral patterns could alter hunting and trapping opportunities (e.g., long-term temperature changes can lead to modifications in animal reproductive cycles, and to alterations in species habitat that can impact species dispersion and concentrations).

Two variables—snowfall and recession/depression years—were not significant in any of the models estimated. Also, recall that earlier preliminary analysis (analyses not shown) rejected the relevance of several other macro-predictors that measured land use patterns and combinations of those land use patterns.

Overall, our results indicated a limited ability to consistently predict trends in hunting for the twelve species over several decades explored in this study using trends in selected macro-level predictors. In general, the hunting trend was best predicted by knowing the level of hunting in the prior year, indicating that the measured external factors have a limited impact on the ability to predict hunting trends. These results suggest the possibility that some unknown process may be driving hunting trends, or that the volume of hunting is somewhat stable to the extent that it is not easily influenced by larger, macro-level forces. Given the persistent hunting-level effect noted across species (the lagged hunting/trapping variable labeled “correction” in Table 1), it is possible that the volume of animals taken by hunting/trapping is better predicted by some excluded variable(s) such as hunting licenses issued. These changes in hunting trends may, therefore, reflect something about “hunting culture” in Wisconsin. Unfortunately, as noted earlier, data on the number of hunting licenses issued in Wisconsin over the long-term of our data was limited.

## 6. Conclusions

Few studies examine whether social, economic, land use, and weather/climate variables can be employed to predict the long-term trend in hunting/trapping. Predicting hunting trends is potentially important for managing wildlife populations. As noted in the related literature, managing wildlife populations is useful because healthy wildlife populations have been shown to be related to ecosystem and human health. The early portion of this manuscript explored those arguments to frame the question of why hunting and animal/ecosystem/human well-being intersections are key considerations.

Our study examined factors affecting the long-term trend in hunting for twelve fur-bearing species in Wisconsin (1930–2018). Our preliminary models (analyses not shown) were based on hypotheses related to changes in land use over time, and included measures of state, federal and park lands, and farmland. These models were inefficient and failed to predict the trends in hunting for any of the twelve species examined, and therefore, land use variables were excluded from further analyses.

Additional models examined pelt and gas prices, snowfall, rainfall, temperature, and urbanization, and included an indicator for recession/depression years. Generally, these variables also proved inefficient in predicting the outcome across both species and time. To be sure, there were some relevant species-specific effects, but those effects were not consistent enough across all species examined to be considered to comprise definitive factors that would create a generalizable model predicting hunting trends that might also have broader policy relevance. For example, both pelt prices and urbanization were significantly related to predicting the hunting trend for five of the twelve species examined, meaning that they had utility for some, but not for most of the species examined. In short, our modeling of these hunting trends indicated that some factors could be used to predict the hunting trend for a specific species. Thus, for example, an increase in urbanization was related to a decline in hunting trends for weasels, bobcats, gray fox and otters. This relationship might well be expected for these species, since they are not typically urbanized species, and urbanization both degrades and fragments habitats suitable for wildlife species [62,63,64].

In terms of policy, our results suggest that one way to modify the volume of hunting and trapping for some species would be to regulate species’ pelt prices. Reflecting a general free market model, higher pelt prices are expected to stimulate species hunting, and it might be tempting to suggest, therefore, that setting maximum pelt prices might limit the hunting and trapping volume for some species. At the same time, however, our models do not measure how price might be related to demand for pelts outside of their effects on hunters/trappers. That is, while it is possible that regulating pelt prices may limit the behavior of hunters/trappers, it also bears mention that at the same time, pelt price regulation may have an opposing effect on demand for pelts by buyers. It is possible, in that scenario, that these opposing tendencies may offset one another. Additional research would be required to examine that possibility and whether hunter/trapper behavior and consumer behavior offset one another. In addition, it should be noted that the pelt price–hunting–pelt-purchasing demand relationship might be quite complex. As pelt prices increase, hunting may increase to provide pelts. Efficient hunting could, however, saturate the pelt market, causing pelt price and perhaps hunting to decline.

Finally, while our study examined factors affecting hunting/trapping trends, it did not address this issue from the perspective of animal rights. The relationship between animal rights and hunting is difficult to address succinctly, and there are many sides to the animal rights debate that are not easily settled outside of a particular philosophical position [65,66,67,68], or in the absence of sufficient research [4]. As a debate between human and nonhuman rights holders, the right to hunt versus the rights of animals to remain free from harm, is—we would suggest—unsolvable philosophically, and because of the philosophical anchoring point in each view, comes down to a belief in one philosophical position over the other. In some places, scientific management of species has resulted in species restoration [69], but while scientific species management policies that reduce animal population may have benefits for ecosystems and human well-being [70], such outcomes fail to address the opposing human–wildlife rights debates (i.e., the human right to hunt versus animal rights to remain free from harm or free from subjection to death by humans), which may be beyond reconciliation. Moreover, the wildlife–human rights debate sometimes overlooks related concerns, such as the survival uses and cultural importance of wildlife hunting by low-income and Native peoples [6,69], which complicates those discussions.

In closing, it should be noted that prior research has also explored hunting as a tool for minimizing human–wildlife conflicts. That latter type of analysis, however, overlooks whether hunting adversely impacts species wellness [70]. Instead of drawing attention to species or environmental wellness, other studies examine the economic benefits of hunting [71,72], which could be argued to be a dimension of human wellness.

## Figures and Tables

**Table 1:** OLS Models predicted number of fur-bearing animals killed by hunting and trapping, Wisconsin, 1930–2018 (BETA/Sig. T; significant effects *p* > 0.05 highlighted).

**Animal Species**									
**Variable**	**Red Fox**	**Beaver**		**Raccoon**	**Skunk**	**Opossum**		**Coyote**	**Otter**
Pelt$	0.055 (0.493)	−0.023 (0.849)		0.285 (0.001)	0.341 (0.001)	0.075 (0.359)		−0.005 (0.945)	0.025 (0.814)
Rain	0.105 (0.201)	0.010 (0.887)		−0.013 (0.816)	−0.036 (0.470)	−0.111 (0.117)		0.006 (0.931)	−0.118 (0.152)
Snow	−0.041 (0.608)	0.092 (0.171)		−0.066 (0.221)	0.031 (0.521)	0.060 (0.395)		−0.092 (0.208)	0.155 (0.059)
Temp	−0.005 (0.955)	−0.041 (0.582)		0.013 (0.822)	0.092 (0.239)	−0.133 (0.096)		0.072 (0.354)	0.001 (0.991)
Rec/Dep	−0.005 (0.950)	−0.032 (0.676)		0.047 (0.423)	0.000 (0.996)	0.037 (0.638)		−0.065 (0.468)	0.035 (0.712)
Gas$	−0.015 (0.853)	−0.107 (0.147)		−0.050 (0.383)	0.023 (0.463)	−0.072 (0.328)		0.192 (0.072)	−0.260 (0.007)
Urban%	−0.135 (0.149)	0.068 (0.601)		0.273 (0.002)	−0.077 (0.333)	0.181 (0.052)		0.127 (0.176)	0.291 (0.020)
Correction	0.754 (0.001)	0.771 (0.001)		0.655 (0.001)	0.573 (0.001)	0.698 (0.001)		0.680 (0.001)	0.450 (0.001)
Constant	--- (0.447)	--- (0.893)		--- (0.107)	---- (0.239)	--- (0.061)		--- (0.228)	--- (0.582)
Adj. R^2^	0.643	0.696		0.807	0.856	0.696		0.684	0.562
N	67	72		71	67	67		64	72
D-W	2.217	1.706		2.304	2.351	2.142		2.215	1.88
**Animal Species**									
**Variable**	**Muskrat**		**Weasels**		**Bobcat**		**Gray Fox**		**Mink**
Pelt$	−0.013 (0.913)		0.331 (0.001)		−0.124 (0.197)		0.329 (0.001)		0.408 (0.010)
Rain	−0.220 (0.066)		−0.082 (0.212)		0.080 (0.344)		−0.008 (0.891)		−0.188 (0.039)
Snow	−0.022 (0.851)		−0.005 (0.940)		−0.090 (0.281)		−0.037 (0.535)		−0.110 (0.221)
Temp	0.017 (0.886)		0.043 (0.556)		−0.144 (0.108)		0.137 (0.046)		0.088 (0.378)
Rec/Dep	−0.123 (0.363)		0.090 (0.232)		−0.055 (0.568)		−0.021 (0.728)		0.090 (0.376)
Gas$	0.001 (0.997)		0.118 (0.228)		0.173 (0.210)		0.511 (0.001)		−0.155 (0.389)
Urban%	−0.247 (0.077)		−0.457 (0.020)		−0.481 (0.012)		−1.184 (0.001)		0.049 (0.782)
Correction	0.200 (0.102)		0.243 (0.050)		0.457 (0.001)		------------		0.277 (0.025)
Constant	--- (0.100)		--- (0.088)		--- (0.002)		--- (0.001)		--- (0.899)
Adj. R^2^	0.080		0.760		0.604		0.771		0.482
N	73		63		63		67		72
D-W	1.883		1.901		2.330		2.336		2.062

The dashes indicate that the Beta coefficient is not estimated in the model.

## Data Availability

All data used in this study are publicly available. Sources for the data are provided in the text of the article.
